# Reliability-Aware Microsystem Design; Compensation for an Ultra-Low-Power Current-Reuse LC-VCO

**DOI:** 10.3390/mi17060713

**Published:** 2026-06-11

**Authors:** Tayebeh Azadmousavi, Ebrahim Ghafar-Zadeh

**Affiliations:** 1 Department of Electrical Engineering, University of Bonab, Bonab 5551395133, Iran; 2 Department of Electrical Engineering and Computer Science, York University, Toronto, ON M3J 1P3, Canada

**Keywords:** current-reuse voltage-controlled oscillator (VCO), reliability, threshold voltage, mobility, phase noise

## Abstract

Aggressive technology scaling has led to a significant increase in manufacturing process variations and transistor aging effects, which critically degrade the performance of radio frequency (RF) circuits. These reliability challenges are particularly pronounced in voltage-controlled oscillators (VCOs), where phase noise and operating frequency stability are compromised. While design strategies incorporating micro-electromechanical systems (MEMS) actuators enhance VCO performance by leveraging MEMS varactors or inductors with substantially higher quality factors (Q), this benefit is progressively undermined over time by process variations and aging-induced shifts in the threshold voltage and carrier mobility of the VCO’s transistors. This work presents an ultra-low-power current-reuse voltage-controlled oscillator (VCO) designed to maintain stable performance under process variability and reliability-induced parameter shifts. Robust operation is achieved using a self-detecting–correcting (SDC) bias scheme that senses performance drift and applies corrective feedback through body-bias control in the VCO core. Analytical relations are derived to describe the impact of threshold voltage and mobility variations, and the approach is validated via post-layout simulations in a 130 nm complementary metal-oxide semiconductor (CMOS). Under 18% variations in threshold voltage and carrier mobility, the proposed SDC scheme preserves oscillation frequency, phase noise, and figure of merit (FoM) while also mitigating the intrinsic output amplitude imbalance of conventional current-reuse VCOs. Monte Carlo analysis (500 runs) demonstrates low sensitivity to fabrication uncertainty, with a standard deviation below 0.14 dBc/Hz for phase noise, 210 kHz for oscillation frequency, and 0.4 dBc/Hz for FoM. The VCO operates from a 0.9 V supply, consumes 175 μW, and achieves −124 dBc/Hz phase noise at 1 MHz offset near 2.4 GHz (FoM ≈ −199 dBc/Hz).

## 1. Introduction

The development of multi-disciplinary complementary metal-oxide semiconductor (CMOS)-based integrated circuits for applications including the Internet of Things (IoT), wireless transceivers, wireless sensor networks, and chemical/biomedical sensors has become an important research area [1,2,3,4,5,6]. The focus in this field converges on portable, implantable, point-of-care (PoC), and lab-on-chip (LoC) systems that are moving toward shrinking device sizes, lower operating voltage, and reduced power consumption. As CMOS technology scales down, the process variability issue increasingly and negatively affects circuit performance, which shows high sensitivity to process, voltage, and temperature (PVT) shifts and stress-induced degradations. Consequently, understanding how process variations affect circuit reliability is indispensable during low-voltage and low-power circuit design. Reliability and process variability challenges, such as negative-bias temperature instability (NBTI), gate dielectric breakdown, and channel hot-electron injection, must be considered in the design of reliable circuits [7,8,9,10,11].

Hot carriers captured at the Si-SiO_2_ interface or inside the oxide layer result in a space charge [12,13]. Over time, charges increasingly accumulate, which leads to trapped charges altering key device characteristics, including an increase in threshold voltage and degradation in transconductance and carrier mobility. HCI accounts for 40–50% of frequency degradation, which directly correlates with mobility reduction [14].

NBTI is an important reliability factor for PMOS transistors, as they typically operate under negative gate bias. NBTI, due to the accumulation of positive interface charges, degrades PMOS characteristics, including currents and gate-drain capacitance; this instability increases the threshold voltage and degrades carrier mobility [15,16]. Based on reported data, NBTI increases threshold voltage by 20–30% over a decade [17]. In advanced nodes from 16 nm to 45 nm, NBTI-induced threshold voltage shift reaches 45.1% after 10 years [18].

High gate voltages can induce gate dielectric breakdown, a time-dependent process caused by strong electric fields across the gate insulator that degrade mobility and increase threshold voltage [7]. As examined above, although HCI and NBTI originate from distinct physical mechanisms (hot carrier injection and gate oxide breakdown, respectively) and occur on different timescales (from hours to years), they all ultimately converge to the same two measurable effects: an increase in threshold voltage and a degradation of carrier mobility [19]. This key observation allows us to model the combined impact of all aging mechanisms, as well as static process variations, using only threshold voltage and carrier mobility as the fundamental variation parameters. Notably, multiple studies have adopted 18–20% threshold voltage and mobility variations to investigate reliability issues [19,20,21,22,23].

The general approach for designing reliable circuits involves incorporating substantial design margins. However, a thorough understanding of the circuit design, considering reliability and process variability, enables optimization of these margins, resulting in better circuit performance. This has attracted considerable research and development in circuit design for reliability methodologies. In [24], the author introduced an on-chip variability sensor based on a phase-locked loop (PLL) to identify different sources of circuit variability. In another design, an adaptive biasing of gate–source voltage to compensate for drain current degradation caused by device reliability mechanisms is developed and employed in an RF power amplifier (PA). In [25], an LC tuning circuit with a variable capacitor for the PA structure is presented that improves efficiency and reliability and mitigates process variation. In another attempt, the DC current of the PA transistor is continuously monitored and compared against a reference value through an operational transconductance amplifier (OTA). In other words, as the transistor’s threshold voltage changes due to reliability issues, the OTA dynamically tunes the DC bias voltage to compensate, ensuring stable PA performance over time. In [26], an adaptive body bias is proposed for PVT sensing of a PA to provide power-added efficiency and output power resilience through threshold voltage adjustment. The reliability of the conversion gain performance of a down-conversion mixer subjected to dynamic stress at elevated supply voltages has been investigated in [27]. The results show a degradation of conversion gain due to the hot electron effect and gate oxide breakdown.

Voltage-controlled oscillators (VCOs) are a key component of many widely used structures such as transceivers [28,29,30,31], frequency synthesizers [32,33,34], VCO-based sensors [35,36,37], and micro-electromechanical systems (MEMS) actuator systems [38,39], which consume most of the available power budget.

For example, low-power VCOs are widely used as core building blocks in CMOS capacitive sensor interfaces, where they translate small capacitance variations into frequency shifts (capacitance-to-frequency conversion). In such architectures, the measured quantity is encoded in the oscillation frequency and subsequently digitized using counters, time-to-digital converters, or VCO-based ADCs. This approach is particularly attractive for compact, low-power sensor nodes because it naturally supports digital-friendly readout and can achieve high resolution when the oscillator exhibits stable gain and low jitter. Representative VCO-based capacitive sensing front ends and mixed-signal readouts have been reported in prior CMOS sensor work [40,41]. In high-precision capacitive sensors, however, the measurement accuracy is often limited by oscillator non-idealities, including phase noise, amplitude imbalance, and long-term drift. Variations in device parameters (e.g., threshold voltage ($V_{th})$ and carrier mobility (μ) can shift oscillation frequency and degrade phase noise, directly translating into bias and uncertainty in the sensor output. Over time, reliability mechanisms can further exacerbate these effects, making robust operation essential for repeatable and reliable sensing, especially in long-term monitoring scenarios. Therefore, compensation techniques that maintain VCO stability under process spread and reliability-induced drift are not only beneficial for RF systems but are also directly relevant to CMOS capacitive sensing platforms that require accurate, reproducible, and low-power measurements [42].

Another important role of the VCO is its significant function in MEMS actuator systems, primarily through integration with MEMS varactors (variable capacitors) and inductors or driving MEMS actuators. MEMS actuators are devices that convert electrical signals into physical motion or mechanical actions at the microscale and play a transformative role in enhancing VCO performance through tunable passive components. In detail, the MEMS actuator physically alters the capacitance value, which, in turn, determines the oscillation frequency of the VCO, enabling precise frequency generation for applications such as wireless communications and RF front-end circuits. On the other hand, MEMS varactors can achieve significantly higher quality (Q) factors that provide lower phase noise [38]. Beyond capacitive tuning, MEMS technology enables alternative approaches to VCO design. Variable inductors using MEMS actuators represent another method for frequency control, potentially offering improved phase noise response and power consumption compared to standard capacitive tuning [39]. As MEMS fabrication advances and new actuation principles emerge, the synergy between MEMS actuators and RF circuits will continue to drive innovation in miniaturized, energy-efficient, and high-performance electronic systems. While the actuator contributes positively to oscillator performance, this improvement is undermined, on the other hand, over time by process variations and aging phenomena, which induce shifts in the threshold voltage and carrier mobility of the VCO’s transistors and degrade the performance. It is worth mentioning that many MEMS actuators, particularly electrostatic comb drives, parallel-plate actuators, and MEMS varactors, require a driving voltage to operate. This driving signal often needs to be a high-frequency AC signal (for resonant actuation) or a precisely controlled DC bias. An LC-VCO is an ideal candidate to generate these signals. An unreliable VCO driving a MEMS actuator will lead to an unreliable system. Conversely, a stable, compensated VCO can mitigate some of the challenges posed by MEMS variability. On the other hand, in applications such as driving a MEMS micro-mirror for a pico-projector in augmented reality (AR) glasses, where battery life is a primary concern, a low-power VCO is required.

Although the design of high-performance VCOs has been carried out for decades [43,44,45], the study and investigation of reliability and process variability in VCO design are continuously attracting much attention among researchers. For example, the hot electron effect on the phase noise degradation of the VCO has been examined. Additionally, in another study, the hot-carrier effects on the VCO’s operation are investigated. After channel hot-electron stress, the phase noise and the frequency operating range are degraded. The authors in [23] discussed the impact of hot carriers and NBTI on the operation of the current-reuse VCO. The results show that these effects significantly degraded the phase noise and tuned oscillation frequency. To the best of our knowledge, no compensation has been reported for current-reuse VCO reliability drift. This paper presents a detecting and correcting circuit with self-bias to mitigate the effects of threshold voltage and mobility variations on the current-reuse VCO’s operation. The introduced circuit effectively overcomes the impact of these process variations and improves the VCO’s stability and robustness. Also, the proposed VCO offers low power consumption, freeing up the system’s power budget for other functions, such as sensing, signal processing, or wireless communication, alongside the MEMS actuation.

This paper proceeds as follows. Section 2 details the proposed self-bias detecting and correcting circuit and derives the analytical equations for investigating reliability issues and compensation processes. Section 3 presents the post-layout simulation results using a 130 nm CMOS process, and Section 4 offers concluding remarks.

## 2. Proposed Circuit Design

**Figure 1:**
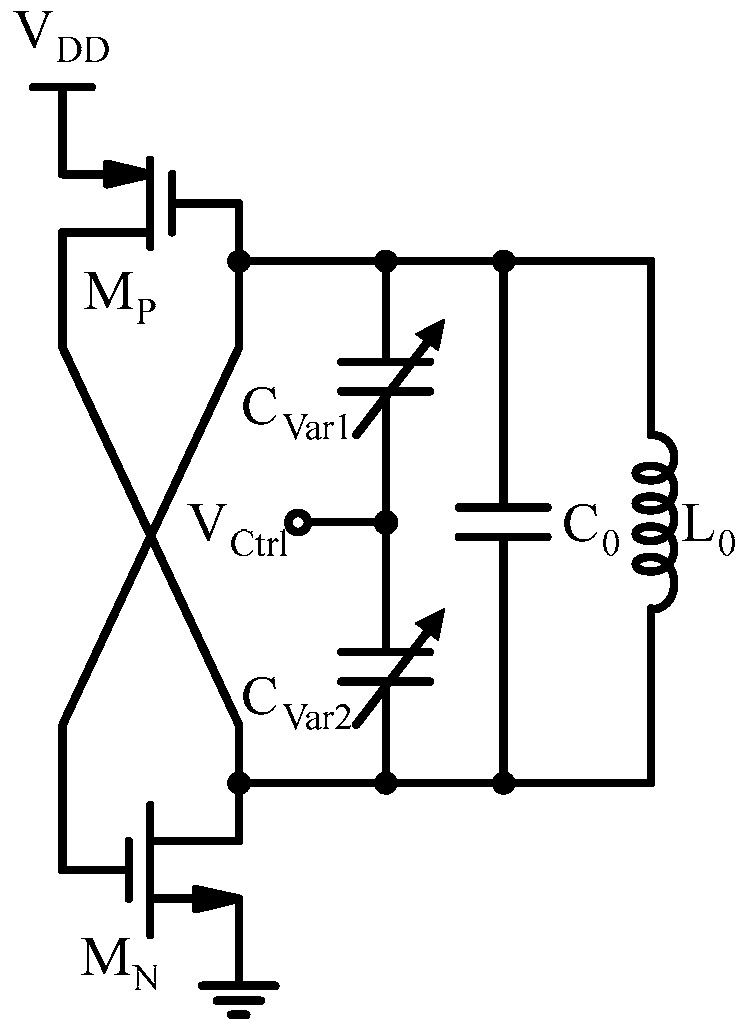
Conventional current-reuse LC-VCO’s structure.

Figure 1 depicts a schematic of the conventional current-reuse VCO. In contrast to the conventional cross-coupled VCOs, where cross-connected transistors switch alternately to generate the negative resistance, the current-reuse VCO operates such that M_P_ and M_N_ are on during the first half cycle and off during the second [45]. This reduces power dissipation by approximately half compared to the typical cross-coupled VCO. This structure, unlike conventional cross-coupled topology, where transistors switch alternately and create a noisy second-harmonic voltage at the common-source node, the current-reuse VCO’s transistors switch simultaneously. This removes the common-source node, making the design inherently immune to phase noise degradation from second-harmonic noise [45,46]. Another source of noise in the oscillator is the tail current source, which is eliminated in the current-reuse structure [47]. So, the current-reuse topology inherently provides better phase noise performance.

However, the asymmetric structure of the current-reuse VCO leads to amplitude imbalance, which is the critical weakness of this configuration. During the first half-cycle, the current-reuse VCO operates in voltage-limited mode, resulting in a smaller voltage swing compared to the second half cycle. This is caused by a large dynamic current passing through MN, leading to transconductance imbalance and, subsequently, non-uniform oscillation voltages [45]. To address imbalance issues, the work in [45] added a degenerative resistance at the source node of the MN transistor. This resistance controls the VCO’s current and constrains the peak dynamic current, ensuring operation in the current-limited region. A key benefit of this technique is providing a symmetric voltage swing across the entire oscillation period. However, the efficacy of this technique is highly sensitive to the accuracy of the degenerative resistance, necessitating precise component values for sufficient imbalance suppression. On the other hand, it increases power consumption and limits the headroom. Additionally, this resistor limits the swing and subsequently degrades phase noise performance [47]. Furthermore, the lack of a tail current source increases sensitivity to PVT variations, necessitating overdesign and ultimately increasing power consumption. Moreover, as mentioned in the previous section, reliability and process variability issues affect VCO performance. To the best of our knowledge, although this issue has been investigated for VCOs, no solution has been provided to resolve the above problem. In this work, we address this issue with a resilient body-biasing technique.

### 2.1. Self-Detecting–Correcting (SDC) Circuit

**Figure 2:**
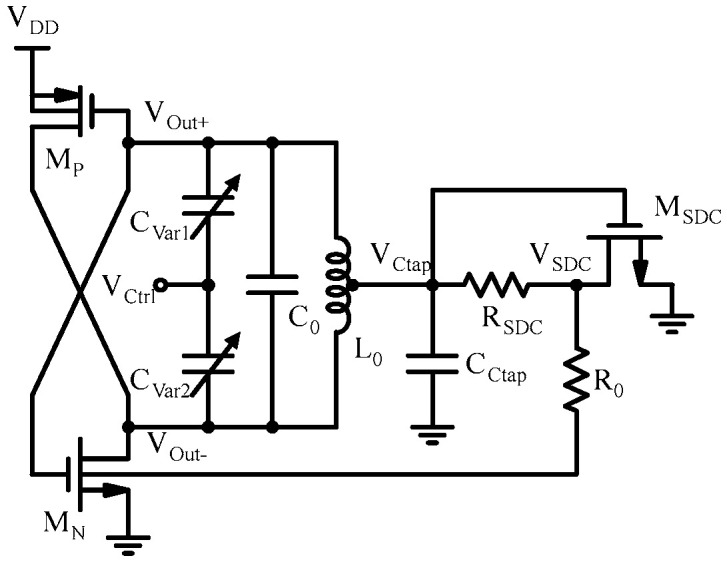
Schematic of the proposed current-reuse LC-VCO with SDC scheme.

The main idea for compensating for reliability issues and process variability is based on detecting these effects on VCO performance using a detection circuit and then applying the generated unwanted effects to the opposite side of the VCO to correct them. The proposed current-reuse VCO with the SDC scheme is shown in Figure 2. A center-tapped inductor is utilized to form the resonant tank with MOS varactors. The tapped node ($V_{Ctap}$) exhibits a voltage with AC and DC components, which is described by Equation (1) [48]. The AC voltage represents the amplitude imbalance voltage between outputs that arises from the difference between the transconductance of M_P_ and M_N_, and the DC component is approximately half of the supply voltage:

(1) $$V_{Ctap}=kV\;Cos\omega t+\frac{V_{DD}}{2}$$

where kV represents the amplitude imbalance. When the oscillation signals are of equal amplitude, the AC components show zero voltage. As shown in Figure 2, a capacitor ($C_{Ctap}$) added to the inductor’s center tap improves amplitude imbalance, which makes the main component of the $V_{Ctap}$ a DC voltage. So, the voltage of $V_{Ctap}$ is utilized for the biasing of the SDC circuit. So, the presented SDC benefits from self-biasing, versus other compensation circuits that require a separate voltage supply for the biasing control circuits [16,25].

As mentioned above, by controlling the current passing through transistor M_N_, the oscillator can operate in the current-limited region during both half cycles, resulting in the improvement of the amplitude imbalance. In the proposed design, a $V_{SDC}$ is applied to the bulk of M_N_, which controls the threshold voltage and, consequently, the current flow through transistor M_N_. Therefore, the SDC scheme effectively improves the unbalanced output amplitude while eliminating the limitations associated with adding degeneration resistance.

It is worthwhile to note that after long-term usage, the transistors of the circuit are affected by the same threshold voltage variability and mobility shifts. When process variability and reliability lead to an increase in $V_{th}$, they degrade the performance of the VCO, such as phase noise, frequency, and output amplitude balance. After that, the voltage of $V_{SDC}$ will be increased. Then, the generated $V_{SDC}$ biases the body terminal of M_N_ to modulate $V_{th}$ and correct the variations. Subsequently, the threshold voltage variation will be compensated, and the VCO can bypass the $V_{th}$ variations and restore transconductance and balance the swing. The same process is validated for carrier mobility compensation. Notably, the resistor of $R_{0}$ as a current-limiting element is employed for suppressing signal leakage between the terminals of $V_{SDC}$ and the M_N_ transistor’s body. As mentioned previously, the DC voltage component at the node $V_{Ctap}$ is approximately half of the supply voltage. This voltage is directly applied to the gate–source terminals of the M_SDC_, which provides insight to determine its dimensions. However, the voltage of $V_{SDC}$ must remain below 0.4 V to prevent a forward-biasing of the p-n junction [49]. Therefore, by considering this maximum voltage constraint of $V_{SDC}$, the values of the resistor $R_{SDC}$ and the transistor size of M_SDC_ can be determined. Notably, the quality of the tank (LC), especially the inductor, affects the phase noise. Therefore, the inductor’s dimensions must be chosen in the design to achieve a high quality factor while simultaneously satisfying the start-up oscillation condition [47].

**Figure 3:**
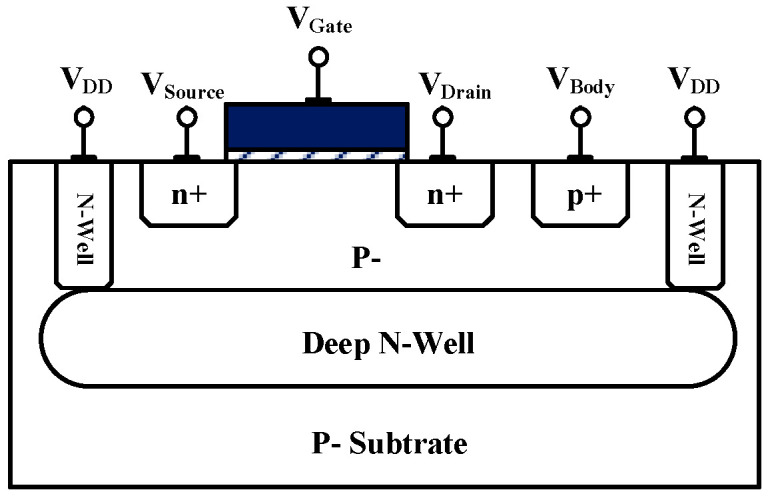
Cross-section of the NMOSFET with DNW.

It is worth mentioning that, in RF circuit design, particularly at frequencies above 1 GHz, it is common practice to use isolated NMOS transistors implemented with a deep N-well (DNW) structure on a P-substrate. The cross-section of the DNW process is illustrated in Figure 3. In this architecture, a DNW is first formed inside the P-substrate, followed by a P-well constructed inside that N-well, within which the NMOS transistor is subsequently built. This configuration effectively isolates the transistor body from the noisy P-substrate, preventing substrate-borne noise from injecting into sensitive RF blocks such as VCOs. Because the body terminal of the NMOS is connected to a potential different from the circuit ground, an additional fabrication process step is required to isolate the NMOS body from the substrate.

An additional key advantage of the DNW structure is that the transistor’s bulk terminal becomes independent of the main P-substrate, allowing the designer to apply a separate body-bias voltage without contaminating the bias node with substrate noise, thereby enabling dynamic threshold voltage adjustment, leakage reduction, or performance tuning in RF circuits as utilized in this work.

While the DNW transistor does occupy a larger area compared to a conventional NMOS device, the increase is not prohibitive. In practice, the area penalty largely depends on the number of isolated body-bias domains and remains within an acceptable range (approximately 10% to 30% of the transistor area), which is justified by the substantial improvements in noise isolation and body-bias flexibility. Concerning fabrication complexity, the DNW requires only one additional mask and one implant step, which makes it a standard option in modern CMOS processes. Regarding compatibility, DNW is widely available in standard CMOS technologies targeting analog, RF, or mixed-signal designs. Therefore, the proposed approach remains practical and well-suited for mainstream CMOS implementation.

**Figure 4:**
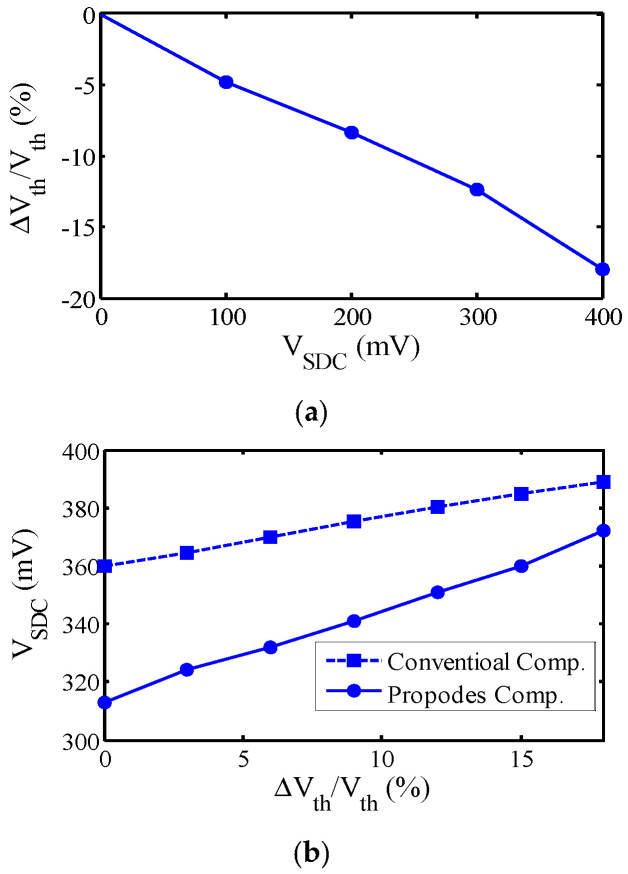
(**a**) Threshold voltage of VCO versus $V_{SDC}$, (**b**) $V_{SDC}$ versus deviation from the threshold voltage of the compensation circuits.

The 18% variation is an assumption based on both the physical limitations of the adaptive body-bias circuit (ensuring that the bulk voltage remains below 0.4 V to avoid forward bias) and the modeling framework adopted in prior reliability studies, which have used similar variation ranges (18–20%) for threshold voltage and mobility. Figure 4a shows that when $V_{SDC}$ is swept as 400 mV, the normalized $V_{th}$ variation (${∆V}_{th}/V_{th}$) of the VCO reaches 18%. On the other hand, Figure 4b shows $V_{SDC}$ versus ${∆V}_{th}/V_{th}$ for the VCO. As can be seen from this figure, when ${∆V}_{th}/V_{th}$ is swept as 18%, $V_{SDC}$ remains below 0.4 V. This limitation of $V_{SDC}$ is also valid for an 18% mobility shift. Therefore, in this design, simulation results are achieved under an 18% shift in both threshold voltage and mobility.

It is worth mentioning that, to provide a comparison with other works, Figure 4b presents the compensation voltage of $V_{SDC}$ for both the presented SDC circuit and the compensation circuit reported in [16]. For the same 18% change in $V_{th}$, the resulting $V_{SDC}$ variation is approximately 60 mV for the proposed circuit and 30 mV for the conventional circuit [16]. This means that the proposed structure is twice as sensitive in translating $V_{th}$ variations into $V_{SDC}$ adjustments. Consequently, it achieves more effective compensation against process and temperature variations in the VCO.

### 2.2. Analytical Equations of Threshold Voltage Variation

This section analyzes the shift in $V_{th}$ resulting from degradation in the transistors of the VCO. The $V_{SDC}$ can be obtained by Equation (2):

(2) $$V_{SDC}=\;V_{Ctap}-R_{SDC}\;\times \;\frac{1}{2}\;\mu_{n}C_{ox}\;{\left(\frac{W}{L}\right)}_{SDC}{\left(V_{Ctap}-V_{th}\right)}^{2}\;\;=\;V_{Ctap}-\frac{{\;K}_{SDC}}{2}{\;R}_{SDC}{\left(V_{Ctap}-V_{th}\right)}^{2}$$

where $K_{SDC}$ includes the MOSFET structure coefficient. From (2), the $V_{SDC}$ shift due to threshold variation is given by (3):

(3) δVSDC=δVSDCδVthδVth = KSDC RSDC VCtap−Vth δVth

To analyze the impact of $V_{SDC}$ voltage on the VCO performance, the start-up requirement for oscillation using the following equation is evaluated:

(4) $$|-(\frac{1}{g_{mN}}+\frac{1}{g_{mp}})|\leq \;R_{Loss}$$

where $g_{mN}$ and $g_{mP}$ represent the transconductance of transistors M_N_ and M_P_, respectively, and the LC tank’s loss is depicted by $R_{Loss}$. In this work, the bulk–source voltage of M_P_ is zero, and the $V_{SDC}$ voltage is applied to the body terminal of M_N_. Therefore, the $g_{mN}$ can be described as:

(5) $$g_{mN}={µ}_{n}\;C_{ox}\;\left(\frac{W}{L}\;\right)\left(V_{GSN}-V_{th}\right)=K_{0}\left(V_{GSN}-V_{th}\right)$$

The $V_{th}$ considering the body effect is well known as Equation (6):

(6) $$V_{th}=V_{th0}+\gamma \left(\sqrt{2\varphi_{f}-V_{BS}}-\sqrt{2\varphi_{f}}\right)=V_{th0}+\gamma \left(\sqrt{2\varphi_{f}-V_{SDC}}-\sqrt{2\varphi_{f}}\right)$$

Equation (6) includes the following parameters: $V_{th0}$ is the threshold voltage at zero bulk–source voltage; γ represents the body effect coefficient, $\varphi_{f}$ is the bulk Fermi potential, and $V_{BS}$ is the voltage applied between the bulk and source terminals. Combining (5) and (6) yields the $g_{mN}$, which can be rewritten as shown in (7):

(7) $$g_{mN}=K_{0}\left(V_{GSN}-V_{th0}-\gamma \left(\sqrt{2\varphi_{f}-V_{SDC}}-\sqrt{2\varphi_{f}}\right)\right)=g_{mN0}-K_{1}\left(\sqrt{2\varphi_{f}-V_{SDC}}-\sqrt{2\varphi_{f}}\right)$$

The overall $g_{mN}$ variation can be described as follows:

(8) δgmN=δgmNδgmN0δgmN0+δgmNδVSDCδVSDC=δgmN0+K1δVSDC22φf−VSDC

where $g_{mN0}$ is the transconductance of transistor M_N_ when $V_{SDC}$ is not applied to the VCO (in other words, the $V_{SDC}$ is zero). The $g_{mN}$ variation arising from the $V_{th}$ shift, obtained by combining (3) and (8), can be described as follows:

(9) $${\delta g}_{mN}=\delta g_{mN0}+K_{1}\;\frac{1}{2\sqrt{2\varphi_{f}-V_{SDC}}}{\;K}_{SDC}{\;R}_{SDC}\;\left(V_{Ctap}-V_{th}\right)\;\delta V_{th}$$

As seen from (9), the process of detecting and correcting the $V_{th}$ variation is accomplished via transconductance equalization. The $\Delta g_{mN0}$ term represents the transconductance shift in M_N_ due to $V_{th}$ variations. The second term compensates for this shift through $V_{SDC}$ thus reducing the transconductance drift resulting from $V_{th}$ degradation. On the other hand, the term of $V_{Ctap}$ in Equation (2) inherently contains the amplitude imbalance arising from unbalanced outputs and, thus, reflects the transconductance drift of both M_N_ and M_P_ transistors. To compensate for these effects, $V_{SDC}$ is applied to the body terminal of M_N_. So, the compensation in the overall performance of the VCO is achieved through the $g_{mN}$ term beside the equalization accomplished by $V_{Ctap}$, which is validated by the simulation results discussed in Section 4.

### 2.3. Analytical Equations of Mobility Variation

The VCO shows performance degradation under mobility drift conditions. Equation (5) demonstrates the impact of mobility variations on the transconductance of the VCO. This effect is mitigated by the SDC circuit, and the analytical relationship is derived and analyzed in this section. Using the expression for $V_{SDC}$ from Equation (2), the fluctuation in $V_{SDC}$ due to mobility degradation can be obtained as:

(10) ΔVSDC=ΔVSDCΔμnΔμn=−12 Cox WLSDCRSDCVCtap−Vth2 Δμn=−K2VCtap−Vth2 Δμn

The fluctuation of transconductance subject to mobility drift using (8) and (10) is expressed as follows:

(11) $${\Delta g}_{mN}={\Delta g}_{mN0}-K_{3}\;\frac{{\left(V_{Ctap}-V_{th}\right)}^{2}}{2\sqrt{2\varphi_{f}-V_{SDC}}}\;\Delta \mu_{n}$$

Mobility variations cause a transconductance shift in M_N_, which is represented by the term $\Delta g_{mN0}$. The term in Equation (11) beyond ${\Delta g}_{mN0}$ represents the compensation for mobility variations. While degraded mobility decreases transconductance, the SDC circuit increases. $V_{SDC}$, which, in turn, leads to an increase in the transconductance of M_N_ and offsets the initial reduction. The derived analytical equations demonstrate the circuit’s ability to overcome both threshold voltage and mobility variations.

## 3. Post-Layout Simulation Results

This section discusses the results of post-layout simulation. The validation presented in this work relies solely on post-layout simulations using foundry-calibrated models for 0.18 μm CMOS technology. While this approach is an industry-standard practice for pre-tapeout design verification, it has inherent limitations. First, simulations cannot capture all the statistical mismatches and second-order effects present in actual silicon, such as random dopant fluctuations, line edge roughness, or thermal gradients across the die. Second, the long-term aging effects (NBTI, HCI) are modeled using empirical equations calibrated to accelerated stress data; these models may not perfectly predict 10-year degradation under real operating conditions with varying temperature, supply voltage, and activity factors. Third, parasitic extraction, while accurate, may miss coupling effects that only appear in physical prototypes. Therefore, while our simulation results strongly indicate the effectiveness of the proposed compensation scheme, silicon measurement remains necessary for final validation. Fabrication and characterization are planned as future work. Importantly, these simulations successfully validated the theoretical and mathematical analyses presented in the previous section.

**Figure 5:**
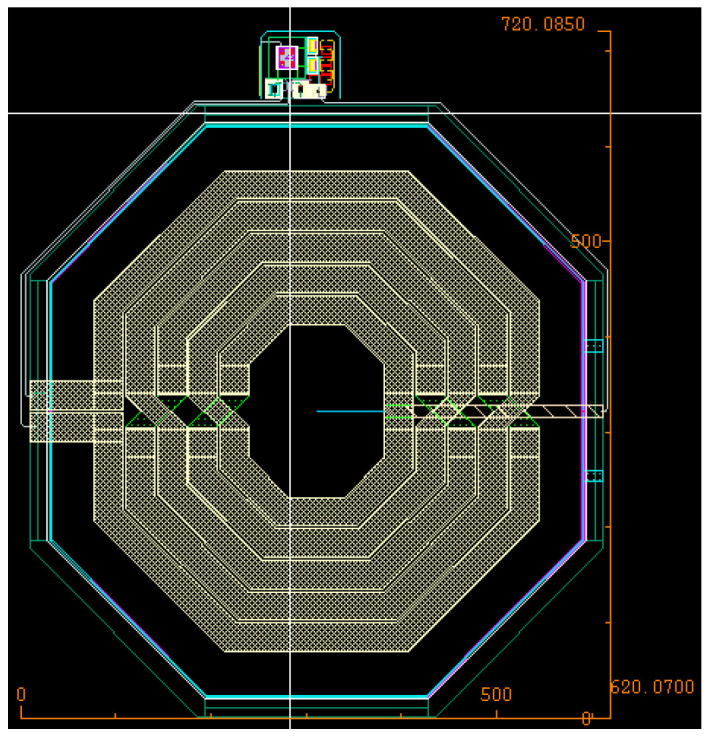
Layout of the designed current-reuse VCO with SDC schema; the occupied area is 720 µm × 620 µm.

**Figure 6:**
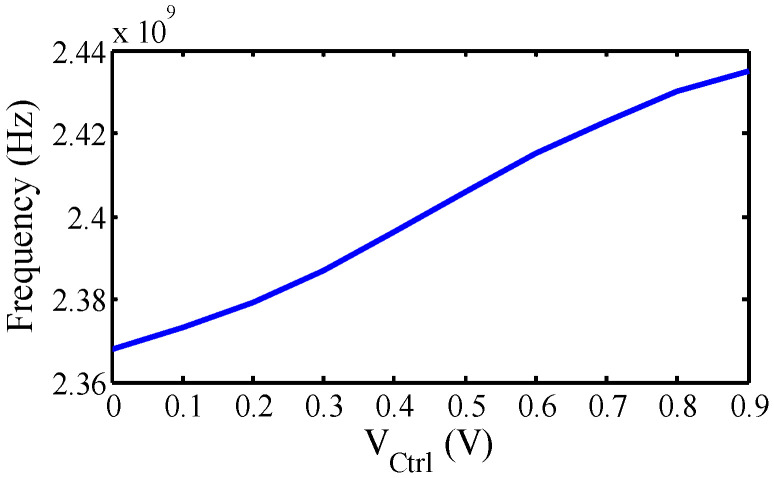
Oscillation frequency versus tuning voltage of the proposed VCO.

**Figure 7:**
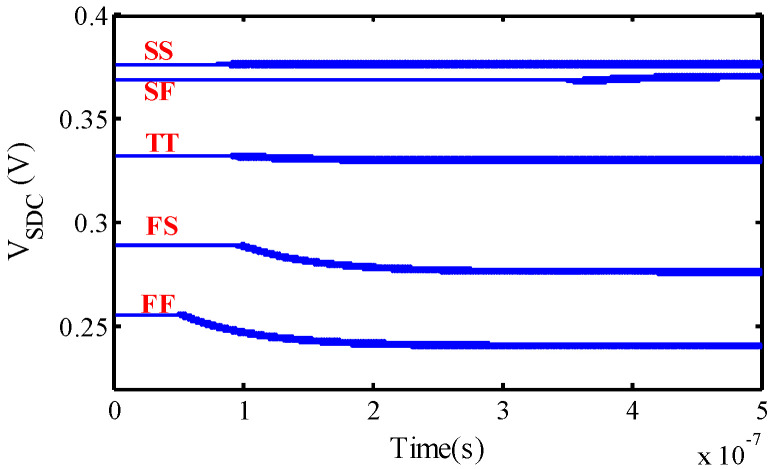
The $V_{SDC}$ voltage at corners of TT, SS, SF, FS, FF.

**Figure 8:**
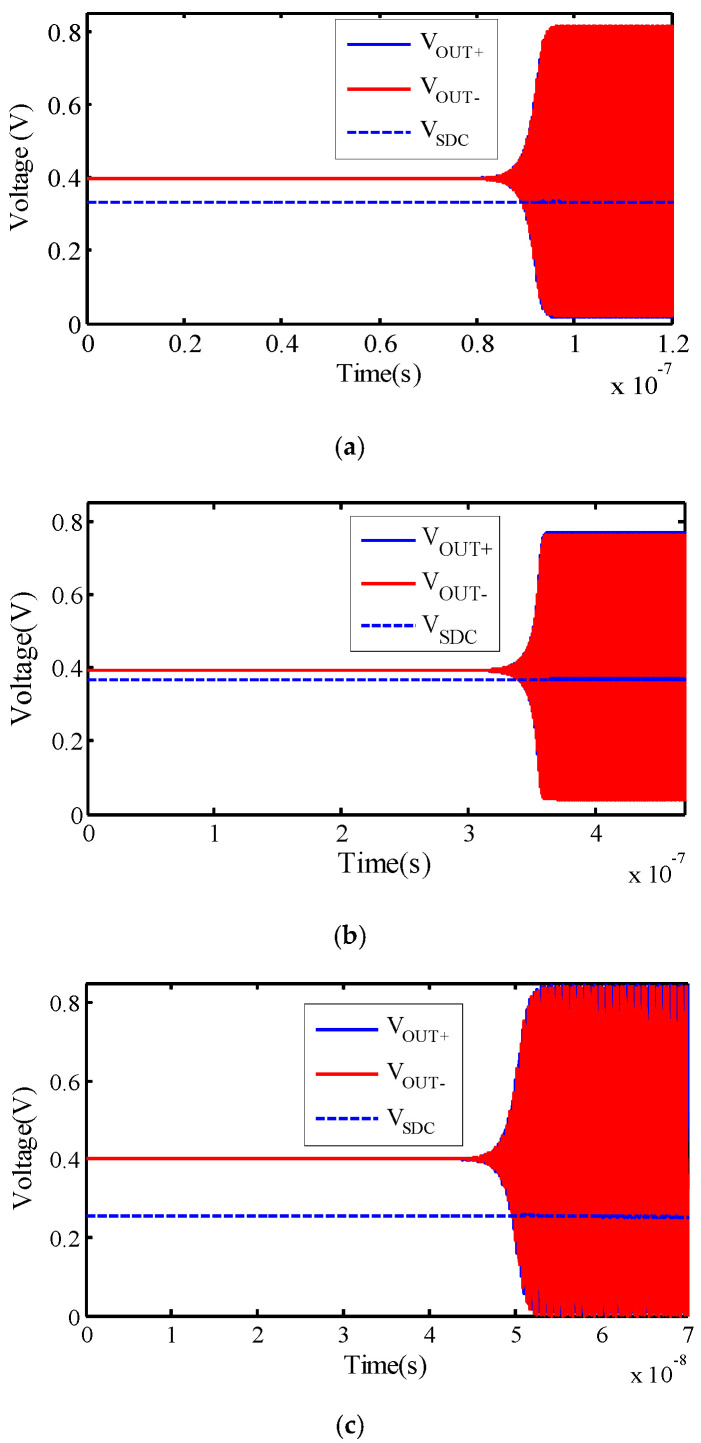
The voltage of $V_{OUT+}$, $V_{OUT-}$ and $V_{SDC}$ at (**a**) TT corner, (**b**) SS corner, (**c**) FF corner.

The presented VCO operates with a 0.9 V supply voltage and consumes 175 µW. The layout view of the proposed current-reuse VCO structure is depicted in Figure 5, and the occupied area is approximately 0.45 mm^2^. The simulated oscillation frequency, as illustrated in Figure 6, is from 2.367 GHz to 2.435 GHz when the tuning voltage is swept from 0 V to 0.9 Vs. The settling behavior of the adaptive bulk voltage was simulated under all relevant process corners (TT, FF, SS, SF, FS). The results shown in Figure 7 demonstrate that the bulk voltage reaches its final value monotonically, with no ringing, no overshoot, and no sustained oscillations in any corner. The settling time is <0.35 µs, confirming a well-damped response. The transient simulation results of the oscillation start-up are shown in Figure 8 for the TT, SS, and FF corners. In the SS corner, the bulk voltage is at its highest value (compensating for low transconductance due to high $V_{th}$), while in the FF corner, it is at its lowest value (preventing excess transconductance and unnecessary power consumption). This variation in bulk voltage ensures that the condition is satisfied across all process corners (TT, FF, SS, SF, FS) without requiring forward body bias (because $V_{SDC}$ < 0.4 V in all cases). We have explicitly verified that in all process corners, the loop gain ($G_{m}\times R_{Loss})$ exceeds unity at the oscillation frequency, guaranteeing reliable start-up (where $G_{m}$ represents the transconductance of the VCO’s transistors). No corner exhibited start-up failure. At steady state, the amplitude of the proposed VCO is constrained by $V_{DD}$ and GND.

**Figure 9:**
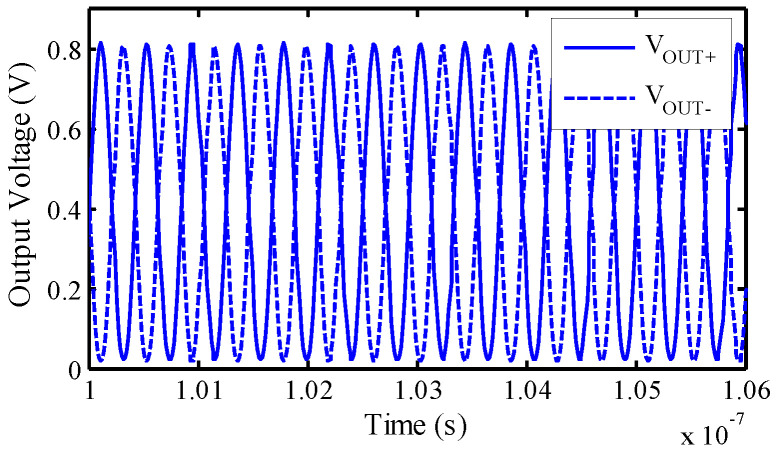
Output voltage waveforms of the proposed VCO that verify the amplitude balancing.

**Figure 10:**
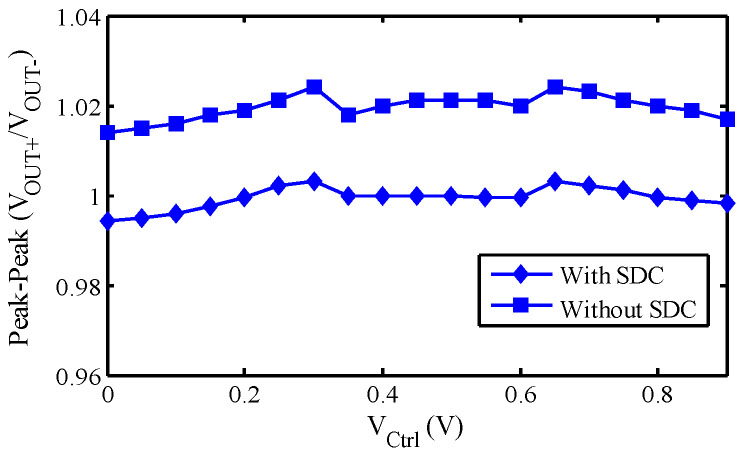
The ratio of the amplitude of $V_{OUT+}$/$V_{OUT-}$ versus control voltage with and without SDC; with SDC, the ratio of $V_{OUT+}$/$V_{OUT-}$ is near 1, which validates the amplitude balancing.

**Figure 11:**
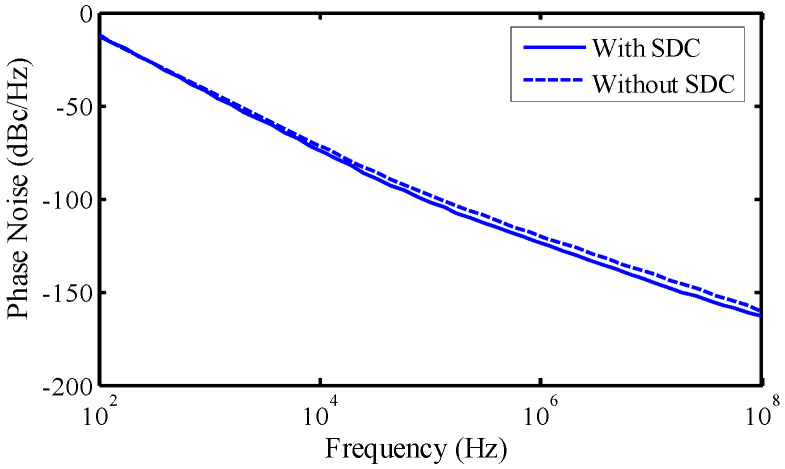
Phase noise of the proposed VCO at an oscillation frequency of 2.4 GHz with and without SDC, showing phase noise improvement of 4 dBc/Hz.

The differential output signals at 2.4 GHz, shown in Figure 9, demonstrate proper amplitude balance. The control voltage ${(V}_{Ctrl}$) is swept, and the output voltage amplitude ratio is plotted in Figure 10. As illustrated, by using an SDC circuit, the amplitude balance across the tuning range is properly preserved. Phase noise is evaluated with and without SDC. As shown in Figure 11, the phase noise at a 1 MHz offset is −124.1 dBc/Hz with SDC and −120.2 dBc/Hz without it. The phase noise improvement can be understood within the Hajimiri–Lee impulse sensitivity function (ISF) framework. In the uncompensated current-reuse VCO, threshold voltage mismatch between the NMOS and PMOS devices leads to asymmetric output swing amplitudes. This asymmetry introduces a non-zero DC component and even harmonics in the ISF, which efficiently upconverts low-frequency 1/f noise—primarily from the tail current source—into close-in phase noise. The proposed body-biasing scheme compensates for the threshold mismatch, restoring amplitude symmetry. As a result, the ISF DC component approaches zero, and even harmonics are suppressed, significantly reducing the upconversion of 1/f noise. Simulation (see Figure 11) indicates a 4 dBc/Hz phase noise improvement at 1 MHz offset.

The figure of merit (FoM) is a key performance metric used for evaluating VCO operation, incorporating phase noise, oscillation frequency, and power consumption into a unified comparative measure. It is defined by the following expression (12):

(12) $$FoM=L\left(∆f\right)-20\log(\frac{f_{0}}{∆f})+10log\;(\frac{P}{1\;\mathrm{mW}})$$

where $L\left(∆f\right)$ is the phase noise at an offset of ∆f from the oscillation frequency $f_{0}$, and the VCO power consumption is presented by *P*. The FoM of the proposed VCO is −199.276 dBc/Hz.

**Figure 12:**
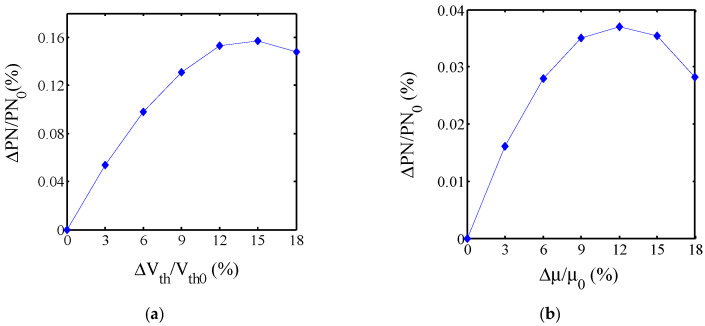
Impact of threshold voltage and mobility variations on oscillator phase noise. (**a**) Normalized phase noise (ΔPN/PN_0_) versus normalized threshold voltage variation (ΔVth/Vth_0_), (**b**) normalized phase noise (ΔPN/PN_0_) versus normalized mobility variation (Δμ/μ_0_). The nominal phase noise (PN_0_) is −124.104 dBc/Hz at 1 MHz offset. Variations are swept from 0% to 18% for both parameters.

**Figure 13:**
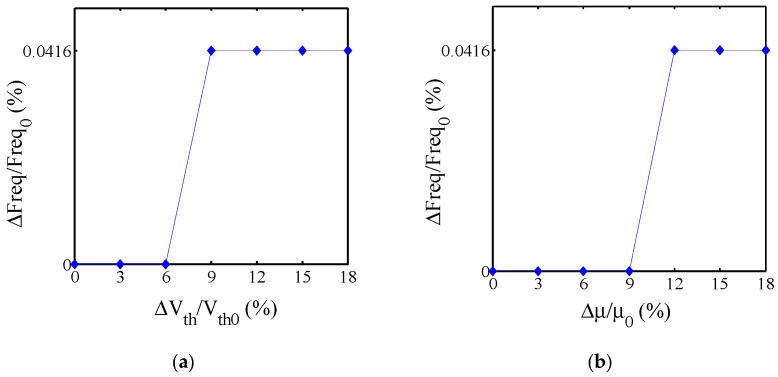
Impact of threshold voltage and mobility variations on oscillation frequency. (**a**) Normalized frequency (ΔFreq/Freq_0_) versus normalized threshold voltage variation (ΔVth/Vth_0_), (**b**) normalized frequency (ΔFreq/Freq_0_) versus normalized mobility variation (Δμ/μ_0_). The nominal frequency (Freq_0_) is 2.4 GHz. Variations are swept from 0% to 18% for both parameters.

It is worth mentioning that the simulation results of the introduced VCO demonstrate that without the SDC scheme, the VCO fails to oscillate at the SS corner, as well as under the mobility and threshold voltage variations. By contrast, the SDC scheme achieves reliable oscillation across all process corners. To verify the effectiveness of the SDC circuit, the threshold voltage and mobility (electron and hole) are varied to analyze their impact on the normalized phase noise (ΔPN/PN_0_), normalized oscillation frequency (ΔFreq/Freq_0_), and normalized FoM (ΔFoM/FoM_0_). Figure 12 shows that for an 18% normalized threshold voltage (Δ$V_{th}$/$V_{th0})$ and mobility shift (Δμ/$\mu_{0}$), the proposed VCO exhibits a normalized phase noise sensitivity of 0.148% and 0.028%, respectively. These results show that the phase noise under threshold voltage and mobility variation is not degraded and even shows slight improvement, indicating no degradation due to reliability issues, which is an outstanding feature provided by the SDC scheme. The oscillation frequency under threshold voltage and mobility shift is shown in Figure 13. With an 18% normalized threshold voltage and mobility shift, the frequency variation is only 0.041%, demonstrating a stable frequency under process variations.

**Figure 14:**
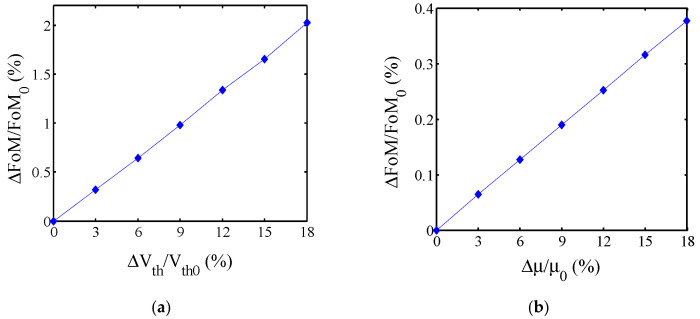
Impact of threshold voltage and mobility variations on FoM. (**a**) Normalized FoM (ΔFoM/FoM_0_) versus normalized threshold voltage variation (ΔVth/Vth_0_). (**b**) Normalized FoM (FoM/FoM_0_) versus normalized mobility variation (Δμ/μ_0_). The nominal frequency (FoM_0_) is −199.276 dBc/Hz at 1 MHz offset. Variations are swept from 0% to 18% for both parameters.

The normalized FoM improves by 2.02% and 0.037% under 18% variations in threshold voltage and mobility, respectively, as shown in Figure 14. This improvement is attributed to stable phase noise and operating frequency, as well as decreasing power consumption with increasing threshold voltage and mobility.

**Figure 15:**
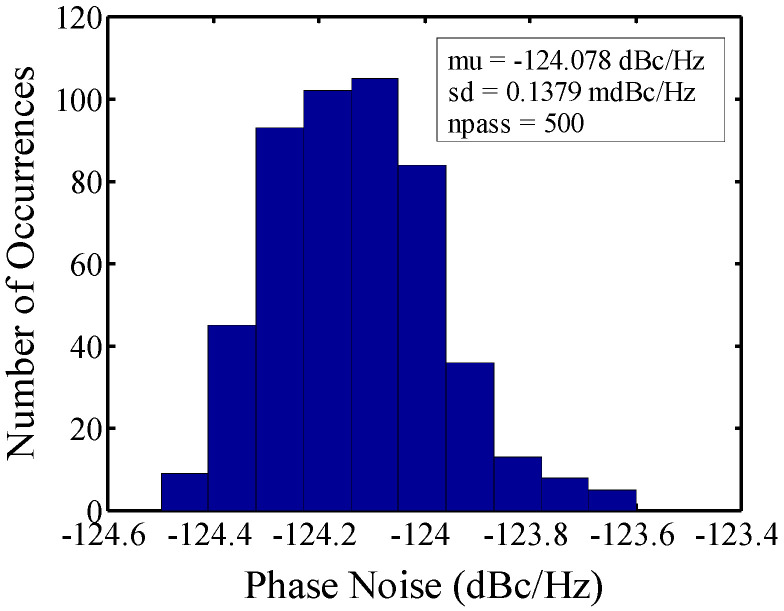
The 500-run Monte Carlo simulation of phase noise.

**Figure 16:**
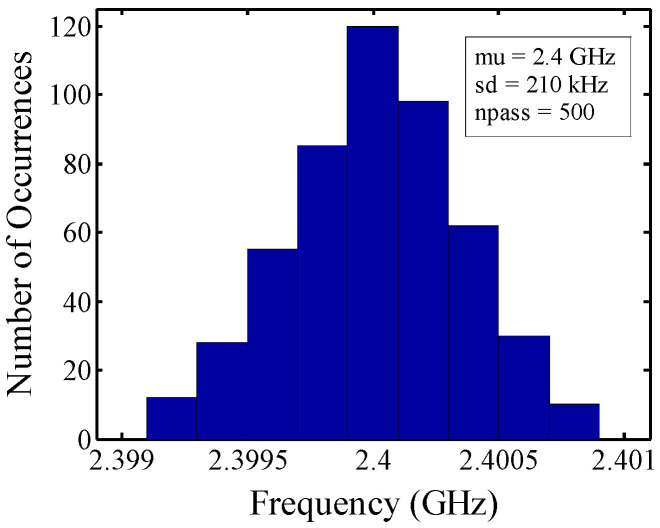
The 500-run Monte Carlo simulation of oscillation frequency.

**Figure 17:**
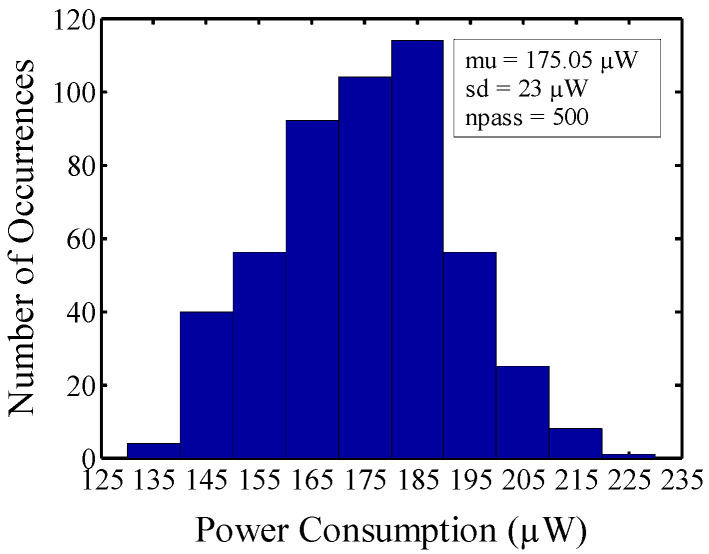
The 500-run Monte Carlo simulation of power consumption.

**Figure 18:**
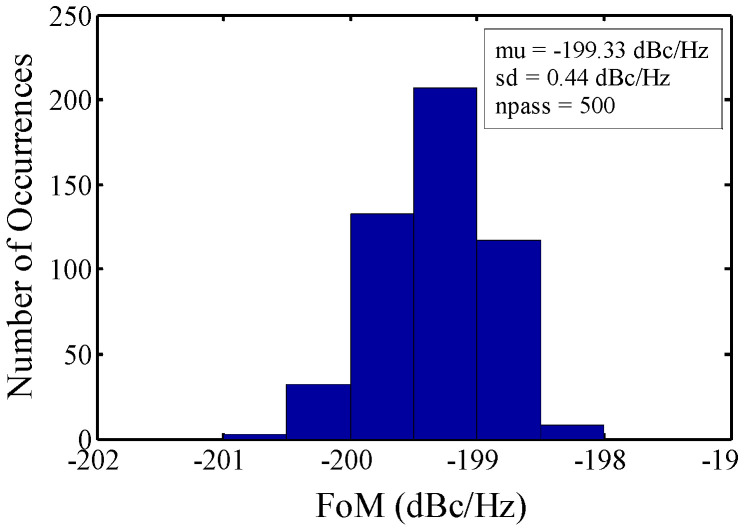
The 500-run Monte Carlo simulation of FoM.

Notably, a well-established principle in oscillator design is that any instability in the loop gain directly corrupts the phase noise spectrum, manifesting as spurious tones or excessive 1/f^3^ noise. To rigorously verify stability, we performed extensive phase noise simulations using a 500-run Monte Carlo analysis considering process and mismatch statistical variations (see Figure 15). The phase noise variation at a 1 MHz offset is < 0.14 dB, with no oscillation dropout or start-up failure in any run, confirming the unconditional stability of the loop gain. Additionally, the proposed VCO’s tolerance to process variations was evaluated through Monte Carlo simulations for oscillation frequency, power consumption, and FoM, as shown in Figure 16, Figure 17, and Figure 18, respectively. The standard deviations are 210 kHz for oscillation frequency, 23 µW for power consumption, and 0.44 dBc/Hz for FoM.

These results confirm the proposed VCO’s low sensitivity to process variations. The VCO’s design parameters are provided in Table 1. The performance comparison between the proposed design and other works is summarized in Table 2, which highlights the proposed VCO’s specifications. Compared to other works, the proposed VCO is able to work under a low supply voltage, consumes low power, and achieves good phase noise performance, resulting in a favorable FoM. Notably, references [50,51] operate at substantially higher oscillation frequencies. In LC-VCOs, the inductor is known to dominate the total chip area, with its area scaling inversely with the square of frequency. Consequently, achieving a lower oscillation frequency inherently demands a larger inductor to preserve a sufficient quality factor, thereby directly increasing the overall area. Meanwhile, the DNW structure—while providing effective body-bias control and enhanced noise isolation—incurs considerable area overhead relative to conventional transistors [52].

## 4. Conclusions

A compensated current-reuse LC-VCO employing an SDC body-bias scheme is proposed to maintain stable operation under threshold-voltage and mobility variations. The operating principle is supported by analytical derivations and validated through post-layout simulation in 130 nm CMOS, including PVT and 500-run Monte Carlo analysis. The proposed scheme mitigates reliability-induced parameter drift while also improving the output-amplitude balance inherent to current-reuse topologies. The VCO operates from a 0.9 V supply, consumes 175 μW, and achieves −124 dBc/Hz phase noise at 1 MHz offset near 2.4 GHz, corresponding to a FoM of approximately −199 dBc/Hz.

## Figures and Tables

**Table 1:** Design parameters of the VCO.

Parameter	W_P_/L_P_	W_N_/L_L_	W_SDC_/L_SDC_
**Value**	6 × 4/0.13 (µm)	5 × 2/0.145 (µm)	1.5/0.170 (µm)
**Parameter**	**L_Ctap_**	**C_Ctap_**	**C_0_**
**Value**	10.87 nH	1.99 pF	0.316 pF
**Parameter**	**R_SDC_**	**R_0_**	
**Value**	8 kΩ	18 kΩ	

**Table 2:** A performance comparison with recently published VCOs.

Work	[45]	[50]	[53]	[51]	[54]	[52]	[22]	[55]	This Work
**CMOS Process (nm)**	180	65	130	65	130	180	65	180	130
**Supply Voltage (V)**	1.25	1.1	1.2	1	1.2	1.8	1	1.8	0.9
**Power Cons. (mW)**	1	1	9.5	0.69	9.6	3.5	2	~12 ^d^	0.175
**Area (mm^2^)**	-	0.046	0.53	0.08	1.12 ^c^	0.12	-	-	0.45
**Frequency (GHz)**	2	12	3.4	5	2.5	2.5	24.29	2.4	2.4
**Phase Noise (dBc/Hz)@1 MHz**	−103 ^a^	−105.3	−136.8 ^b^	−126.7 ^b^	−125.63	−123.5	−88.34	~−117 ^d^	−124.104
**FoM (dBc/Hz)**	−189.3	−196.3	−188.1 ^b^	−192.8 ^b^	−183.77	−185.9	−173 ^d^	−173 ^d^	−199.276
**Compensation Range**	-	Vth	-	-	-	-	Vth	Vth and µ	Vth and µ
**Variation Tolerance (%)**	-	-	-	-	-	-	21	~25 ^d^	18
**Maximum Variation in Phase Noise (%)**	-	-	-	-	-	-	3.8 ^d^ @ Vth	0.85 ^d^ @ Vth 0.85 ^d^ @ µ	0.16 @ Vth 0.036 @ µ

^a^ at 100 KHz offset, ^b^ at 3 MHz offset, ^c^ with pads, ^d^ estimated.

## Data Availability

The original contributions presented in this study are included in the article. Further inquiries can be directed to the corresponding authors.
